# A randomized phase III study of pretransplant conditioning for AML/MDS with fludarabine and once daily IV busulfan ± clofarabine in allogeneic stem cell transplantation

**DOI:** 10.1038/s41409-022-01705-7

**Published:** 2022-05-24

**Authors:** Borje S. Andersson, Peter F. Thall, Junsheng Ma, Benigno C. Valdez, Roland Bassett, Julianne Chen, Sairah Ahmed, Amin Alousi, Qaiser Bashir, Stefan Ciurea, Alison Gulbis, Rita Cool, Jitesh Kawedia, Chitra Hosing, Partow Kebriaei, Steve Kornblau, Alan Myers, Betul Oran, Katayoun Rezvani, Nina Shah, Elizabeth Shpall, Simrit Parmar, Uday R. Popat, Yago Nieto, Richard E. Champlin

**Affiliations:** 1 Department of Stem Cell Transplantation and Cellular Therapy, The University of Texas M.D. Anderson Cancer Center, Houston, TX USA; 2 Department of Biostatistics, The University of Texas M.D. Anderson Cancer Center, Houston, TX USA; 3 Department of Pharmacy, The University of Texas M.D. Anderson Cancer Center, Houston, TX USA; 4 Present Address: University of California, San Francisco, CA USA

**Keywords:** Phase III trials, Chemotherapy

## Abstract

Pretransplant conditioning with Fludarabine (Flu)-Busulfan (Bu) is safe, but clofarabine (Clo) has improved antileukemic activity. Hypothesis: Flu+Clo-Bu (FCB) yields superior progression-free survival (PFS) after allogeneic transplantation. We randomized 250 AML/MDS patients aged 3–70, Karnofsky Score ≥80, with matched donors, to FCB (*n* = 120) or Flu-Bu (*n* = 130), stratifying complete remission (CR) vs. No CR, (NCR). HCT-CI scores varied, from 0 to 10. All evaluable patients engrafted. Median follow-up was 66 months (interquartile range: 58–80). Three-year relapse incidence (RI), 25% with FCB, vs. 39% with Flu-Bu (*p* = 0.018), offset by higher non-relapse mortality, 22.6% (95%CI: 16–30.2%) vs. 12.3% (95%CI: 6.5–19%). Three-year PFS was 52% (95%CI: 44–62%) (FCB), vs. 48% (95%CI: 41–58%) (Flu-Bu). FCB benefited CR patients less, NCR patients age ≤ 60 had 3-year 34% RI (95%CI: 19–49%) (FCB) vs. 56% (95%CI: 38–70%) after Flu-Bu (*p* = 0.037). NCR patients >60 years had 3-year RI 10.0% (FCB), vs. 56.0%, after Flu-Bu (*p* = 0.003). Bayesian regression analysis including treatment-covariate interactions showed FCB superiority in NCR patients with low HCT-CI (0–2). Serious adverse event profiles were similar for the regimens. Conditioning with FCB did not improve PFS overall, but improved disease control in NCR patients, mandating confirmatory trials. Remission status and HCT-CI should be considered when using FCB.

## Introduction

The conditioning regimen used in allogeneic stem cell transplantation (allo-SCT) is important for long-term outcomes, due to the need for potent antileukemia activity and sufficient immunosuppressive effect to allow engraftment, rebuilding the host’s immune system, and establishing a graft-versus-leukemia effect. For AML patients, conditioning treatment recently shifted from a double alkylator-based regimen (Busulfan-Cyclophosphamide) [1, 2] to a nucleoside analog (NA), fludarabine (Flu), combined with an alkylating agent, IV Busulfan (Bu) [3–6]. Factors contributing to the safety and efficacy of Flu-Bu include nonoverlapping toxicities due to alternative routes of metabolic disposition, and the predictability of Bu systemic exposure (Bu-SE), especially when used with therapeutic dose monitoring [7, 8].

Model studies in human AML cell lines demonstrated significant synergy when one, or preferably two NAs are combined with Bu, and also between the NAs themselves. This synergy is further enhanced by Bu, but optimized sequencing and timing of the agents are required, such that synergistic rather than additive or even antagonistic effects are achieved [9, 10]. Based on such in vitro studies, a later generation NA Clofarabine (Clo), was explored with Flu and Bu in pretransplant conditioning of high-risk AML patients [11]. This study established that a double NA regimen had an acceptable toxicity profile, was efficacious, and that higher Clo doses yield greater antileukemic effects. The preferred dose levels were Flu at 10 mg/m^2^ and Clo at 30 mg/m^2^, combined with Bu in a modification of our previously reported Flu-Bu regimen [4]. We then hypothesized, that Flu+Clo-Bu (FCB) would be superior to Flu-Bu, especially in high-risk AML patients with detectable disease/not in complete remission (NCR). This led us to conduct a randomized phase III study comparing FCB with our standard Flu-Bu regimen as conditioning for AML/MDS patients undergoing allo-SCT.

This report presents statistical analyses of mature follow-up data from 250 patients randomized to FCB or Flu-Bu. Patients above age 60 received a lower targeted Bu-SE dose than younger patients, per our departmental standard.

## Subjects and methods

### Patient eligibility

This trial included AML patients in first CR (CR1), with high-risk features, defined as cytogenetics other than translocation (*t*)(8;21), inversion (inv)16, or *t*(15;17), and/or the need for more than one cycle of chemotherapy to achieve CR. Complete remission was defined with standard cytological criteria, i.e., less than 5% BM blasts, 1 × 10^9^ granulocytes/L, and 100 × 10^9^ platelets/L. Any AML patient who had >5% blasts in the BM and/or circulating blasts or with documented extramedullary disease was considered to have active disease/NCR. Cytogenetic risk categories were based on the ELN classification [12, 13]. Patients with induction-chemotherapy refractory AML, or disease beyond CR1 were also eligible. For MDS patients, eligibility allowed an International Prognostic Score System score of ≥2 [14, 15], or progression after previous chemotherapy. Eligibility required age 3–70 years, acceptable renal (creatinine ≤ 1.5 mg/dl), and hepatic function (bilirubin and SGPT ≤ 2 times the upper normal limit); Karnofsky performance status (KPS) ≥ 80; no uncontrolled infection, negative serology for hepatitis B, -C, and HIV; adequate cardiac function (LVEF ≥ 40%), and pulmonary function (FEV1, FVC, and DLCO ≥ 50% of predicted). At the time of study design, it had not been determined whether hematopoietic cell transplantation-specific comorbidity score (HCT-CI) would influence outcomes if the patient’s KPS and normal organ function assessments were acceptable [16]. Eligibility thus included patients with any HCT-CI, if they had acceptable organ functions and a satisfactory KPS.

Stem cell grafts were obtained from human leukocyte antigen (HLA)-compatible related (10/10 matched) donors or matched unrelated donors (MUDs). HLA-matching was assessed using high-resolution DNA-typing. Engraftment and T-cell chimerism from blood and marrow was documented with PCR-based technology, and presence of 1–99% host cell DNA in post-transplant samples was considered mixed chimerism [17]. Adult patients provided written informed consent, the minors assented, their consent was given by parental caregivers in keeping with institutional guidelines and the declaration of Helsinki.

### Pretransplant conditioning program

Treatments were based on the 4-day Flu-Bu regimen of de Lima [4], modified from Russell [3]. For FCB, Fludarabine (10 mg/m^2^) (Fludara^®^, Genzyme Corporation, Cambridge, MA) was infused over 60 min once daily for 4 days (days −6 to −3), each dose followed by Clo (Clolar^®^, 30 mg/m^2^, Genzyme Corp.), over 60 min, then Bu (IV Busulfex^®^ [busulfan] Injection, Otsuka America Pharmaceuticals Inc., Princeton, NJ), over 3 h, all through a central line by controlled-rate pump. Busulfan was targeted to an average daily Bu-SE, represented by the area under the concentration vs. time curve (AUC) of 6000 µMol-min, total course AUC 24,000 µMol-min ± 5% for patients up to age 60. For patients ages 61–70, the targeted daily Bu-SE was 4000 µMol-min, total course 16,000 µMol-min ± 5%. Pharmacokinetic parameters from a Bu “test dose” of 32 mg/m^2^ administered 2–7 days before conditioning were used to calculate the therapeutic dose [8]. For Flu-Bu, Flu at 40 mg/m^2^ was administered IV over 60 min once daily, days −6 to −3, each dose followed by the same targeted Bu dose described above for FCB. Stem cells were infused on day 0.

Graft-vs-host disease (GVHD) prophylaxis was tacrolimus/mini-methotrexate [18]. Additionally, 4 mg/kg of rabbit antithymocyte globulin (Thymoglobulin^®^, Genzyme Corp.) was administered to MUD recipients on days −3 to −1 [4, 11].

### Data collection

Clinical data were collected from 250 patients with AML or MDS, with 120 patients randomized to receive FCB and 130 Flu-Bu. Patients with MDS/AML (*n* = 22) and MPD/AML (*n* = 1) were classified as AML. Patients in CR1 or CR2 were classified as CR, all others were classified as NCR. Toxicities were assessed daily while in-patients, using the NIH common terminology criteria vs. 3.0 [19]. Veno-occlusive disease (VOD/SOS) was assessed using McDonald’s criteria, as modified by the Johns Hopkins group [20, 21]. Acute and chronic GVHD (aGVHD/cGVHD) were graded using the Keystone- and NIH criteria, respectively [22, 23]. For aGVHD maximum grade, patients with grade 0 or 1 aGVHD were categorized as one group. Age was summarized as a continuous variable, and also dichotomized using the cutoff of 60 (age ≤ 60, versus age >60). HCT-CI scores were dichotomized as 0–2 vs. 3–10. The trial was registered with the US National Cancer Institute on ClinicalTrials.Gov, Identifier NCT 01471444.

### Statistical methods

The primary outcome was progression-free survival (PFS) time, from transplant to date of progressive disease (PD) or death, with patients alive without PD at last follow-up administratively censored. The randomization was stratified by blocking disease activity (CR/NCR), using a three-stage group sequential design with overall type I error rate 5% and power 90% to detect a difference in median PFS from 14.35 to 25.29 months. Non-relapse mortality (NRM) and relapse times were analyzed as competing risks and compared using Gray’s test [24]. Unadjusted PFS and overall survival (OS) time distributions were estimated by the Kaplan–Meier (KM) method [25] and compared using the log-rank test [26]. Median follow-up times were calculated using the reverse KM method. Bayesian piecewise exponential (PE) regression models were fit to estimate prognostic effects on PFS and OS time of the covariates age (≤60 vs. >60), disease type (AML vs. MDS), patient cytogenetic risk category by the ELN-classification (intermediate/good vs. poor) [12, 13], comorbidities/HCT-CI score [16], cell type (HPC-A vs. HPC-M), CR (CR vs. NCR), and donor relation (unrelated vs. sibling) [27]. Because age, disease status, and HCT-CI score are well established prognostic covariates, based on preliminary goodness-of-fit analyses, models for PFS and OS were fit with age dichotomized as Age60 = [age ≤ 60] = [younger age], HCT-CI score dichotomized as [good] = 0–2 versus 3–10 = [bad], including the treatment-covariate interactions FCB × Age60, FCB × CR, and FCB × HCT-CI. Model fits with three HCT-CI subgroups, 0–2, 3–5, and 6–10 were very similar to fits with the two subgroups 0–2 and 3–10. In all Bayesian models, non-informative priors were assumed for all parameters. The number of constant hazard subintervals in the PE model was set to eight with each subinterval containing approximately the same number of events.

To interpret each fitted Bayesian regression model, the posterior probability Pr(*β* < 0 | Data) is the probability of a beneficial effect (PBE) for the coefficient *β* of a covariate or indicator that *β* multiplies in the model’s linear term. Larger values of PBE, above 0.90 or 0.95, correspond to a higher likelihood of the covariate reducing the hazard, corresponding to longer average PFS or OS time. Smaller PBE values, below 0.10 or 0.05, correspond to the covariate being likely to have a harmful effect on the outcome, with shorter average PFS or OS time. All statistical analyses were performed using R-v 3.6.1 and SAS 9.4. Convergences were monitored using trace plots and the Gelman-Rubin Diagnostics statistic (<1.1 indicating convergence) [28].

Summary statistics for baseline patient covariates (gender, race, age, disease type/diagnosis, treatment-related AML and MDS, cytogenetic risk category, total chemotherapy regimens, number of prior autologous transplants, CR/NCR, KPS, cell type, Bu-SE, allotype, donor relation, and HCT-CI score) were computed for all patients and within each treatment arm. Tabular summaries for aGVHD/cGVHD and engraftment covariates were computed similarly. Categorical covariates were summarized by frequencies and percentages. Continuous covariates were summarized by medians, standard deviations, minimum and maximum.

## Results

### Patient characteristics

Two hundred fifty patients with AML (*n* = 181), and MDS (*n* = 69) received allo-SCT on this protocol between December 5, 2011 and September 30, 2015. Median age at transplant was 51.4 years (range, 8–70), 71 patients (28.4%) were >60 years, two were pediatric (8 and 13 years, randomized to Flu-Bu), 109 (43.6 %) were female, 141 (56.4%) were male. One hundred fifty-five subjects (62.0%) received MUD SCTs, 95 (38.0%) received grafts from matched related donors; 85 (34.0%) patients received marrow (BM), and 165 (66.0%) peripheral blood progenitor cells. Two hundred thirteen (85.5%) had poor or intermediate-risk cytogenetics [12, 13], 133 (53.2%) were in cytologic CR and 117 (46.8%) had active disease (NCR) at conditioning, defined as >5% BM blasts and/or circulating blasts. Pretransplant patient characteristics are summarized in Table 1.

**Table 1:** Summary of patient baseline characteristics, overall and by treatment group.

Variable	*N*	Overall	Flu-Bu	FCB
Gender, *n* (%)	250			
Female		109 (43.6)	54 (41.5)	55 (45.8)
Male		141 (56.4)	76 (58.5)	65 (54.2)
Race, *n* (%)	243			
Asian		7 (2.9)	4 (3.2)	3 (2.6)
Black		8 (3.3)	5 (4.0)	3 (2.6)
Hispanic		10 (4.1)	8 (6.3)	2 (1.7)
Other		6 (2.5)	3 (2.4)	3 (2.6)
White		212 (87.2)	106 (84.1)	106 (90.6)
Age, Years Median (SD)	250	51.4 (13.5)	50.9 (13.7)	51.9 (13.2)
Range		8–70	8–69	21–70
Age60, *n* (%)	250			
≤60		179 (71.6)	93 (71.5)	86 (71.7)
>60		71 (28.4)	37 (28.5)	34 (28.3)
Diagnosis, *n* (%)	250			
AML		181 (72.4)	95 (73.1)	86 (71.7)
MDS		69 (27.6)	35 (26.9)	34 (28.3)
Tx Related MDS, *n* (%)	69			
No		56 (81.2)	31 (88.6)	25 (73.5)
Yes		13 (18.8)	4 (11.4)	9 (26.5)
Tx Related AML, *n* (%)	181			
No		155 (85.6)	78 (82.1)	77 (89.5)
Yes		26 (14.4)	17 (17.9)	9 (10.5)
Cytogenetic risk category, *n* (%)	249			
Poor		93 (37.3)	54 (41.5)	39 (32.8)
Intermediate/Good		156 (62.7)	76 (58.5)	80 (67.2)
No. Chemotherapy regimens, Median (SD)	250	1.47 (0.85)	1.48 (0.89)	1.46 (0.80)
Prior Autologous TP, *n* (%)	250			
0		247 (98.8)	129 (99.2)	118 (98.3)
1		2 (0.8)	1 (0.8)	1 (0.8)
2		1 (0.4)	0 (0.0)	1 (0.8)
CR status, *n* (%)	250			
CR		133 (53.2)	69 (53.1)	64 (53.3)
NCR		117 (46.8)	61 (46.9)	56 (46.7)
Karnofsky Performance, *n* (%)	243			
70		3 (1.2)	2 (1.6)	1 (0.8)
80		27 (11.1)	15 (12.0)	12 (10.2)
90		119 (49.0)	66 (52.8)	53 (44.9)
100		94 (38.7)	42 (33.6)	52 (44.1)
Cell type, *n* (%)	250			
^ a^HPC-A		165 (66.0)	85 (65.4)	80 (66.7)
^ b^HPC-M		85 (34.0)	45 (34.6)	40 (33.3)
Bu-SE, AUC, *n* (%)	250			
4000		77 (30.8)	41 (31.5)	36 (30.0)
6000		173 (69.2)	89 (68.5)	84 (70.0)
Allotype/relation, *n* (%)	250			
HLA-identical sibling		95 (38.0)	49 (37.7)	46 (38.3)
Unrelated		155 (62.0)	81 (62.3)	74 (61.7)
‐ Match 10/10		151 (60.4)	81 (62.3)	70 (58.3)
‐ Match 9/10		4 (1.6)	0 (0.0)	4 (3.3)
HCT-CI score, *n* (%)	250			
0–2		107 (42.8)	56 (43.1)	51 (42.5)
≥3–10		143 (57.2)	74 (56.9)	69 (57.5)

^a^Peripheral blood progenitor cells were obtained through cytapheresis.

^b^Bone marrow progenitor cells.

### Clinical outcomes

All evaluable patients engrafted (*n* = 248) at a median of 12 days (range, 10–25), with two FCB patients inevaluable (early deaths). Full donor chimerism was achieved in 221 (89.1%) patients, 92.4% of the FCB group, and 86.2% of the Flu-Bu group. Two hundred forty (97.6%) evaluable patients remained in or achieved CR following transplant. One hundred eleven patients (44.8%) developed grade II–IV aGvHD, while 16 (6.5%) developed grade III–IV aGVHD, 93 (39.4%) developed cGVHD (Supplement Table S1). No unexpected renal, hepatic, or neurologic toxicities were seen, and there were no significant differences in the toxicity profiles between Flu-Bu and FCB (Table 2). Twenty-six FCB patients died of non-relapse causes, 13 with age ≤60, 13 with age >60; one patient in each age category died of cGVHD in the HCT-CI 0–2 subgroup. In the HCT-CI 3–10 group, 12 patients in each age category died of NRM causes, most commonly infections and GVHD. Seven Flu-Bu patients in the ≤60 age group died of non-relapse causes, most commonly infections, while six patients above age 60 died, with GVHD being the predominant cause. Transient bilirubin elevations were seen to a similar extent and duration in the treatment arms. There was no case of VOD/SOS after Flu-Bu, while three cases were encountered after FCB (Table 2), two of whom had high CTC-CI scores (4 and 5, respectively), and developed grade 3 “late onset” VOD two and 3 months post-transplant in the setting of aGVHD. Both patients recovered after management with diuretics and steroids. The third patient, a female in CR2, HCT-CI 5, started FCB while pancytopenic, 16 days after Flu with high-dose Cytarabine and Idarubicin. She developed VOD and succumbed to multi-organ failure. Finally, there was a higher incidence of serious post-transplant infections after FCB, with six patients dying of bacterial infections vs. one after Flu-Bu (Table 2). Six patients ≤60 years were erroneously targeted to a daily Bu-SE of 4000 µMol-min, and three patients with KPS < 80 were entered in the trial.

**Table 2:** All adverse events by grade and treatment group.

SAE	Flu-Bu (*N* = 130)						FCB_(*N* = 120)					
	Grade 1	Grade 2	Grade 3	Grade 4	Grade 5	ALL	Grade 1	Grade 2	Grade 3	Grade 4	Grade 5	ALL
Mucositis	27	67	25	0	0	119	29	67	19	0	0	115
VOD/SOS	0	0	0	0	0	0	0	0	2	0	1	3
Elevated Bilirubin	10	25	7	0	0	42	10	32	6	1	0	49
PRES	0	0	1	0	0	1	0	0	3	0	0	3
DAH	0	0	0	0	2	2	0	0	0	1	1	2
Pneumonitis	0	0	5	0	1	6	0	0	3	1	1	5
Hand/Foot Syndrome	6	5	0	0	0	11	6	4	1	0	0	11
Infection: Bacterial	13	5	11	3	1	33	3	4	21	3	6	37
Infection: Viral	9	55	4	1	2	71	10	33	9	0	3	55
Infection: Fungal	0	0	2	2	2	6	0	0	4	2	3	9

*SAE* Serious Adverse Events, *VOD* Hepatic Veno-Occlusive Disease/Sinusoidal Obstruction Syndrome, *PRES* Posterior Reversible Encephalopathy Syndrome, *DAH* diffuse alveolar hemorrhage.

**Fig. 1:**
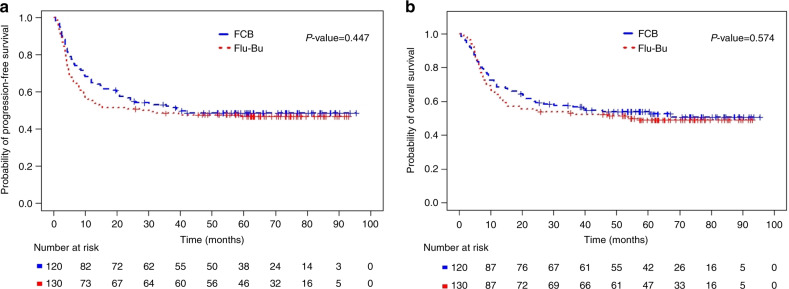
Kaplan-Meier plots of Progression-Free and Overall Survival for all patients. Kaplan–Meier plots of PFS and OS for all patients treated with FCB (*N* = 120) and Flu-Bu (*N* = 130). **a** Kaplan–Meier plots of PFS for all patients treated with FCB (number of PFS events = 61, median PFS 39.3 months) and Flu-Bu (number of PFS events =69, median PFS 28.1 months). *P* value is 0.447, log-rank test. **b** Kaplan–Meier plots of OS for all patients treated with FCB (number of deaths =57, median OS not reached) or Flu-Bu (number of deaths = 66, median OS 53.9 months). *P* value is 0.574, log-rank test.

Median follow-up time for all patients was 66 months (interquartile range, IQR: 58–80). Kaplan–Meier plots of PFS and OS are presented in Fig. 1a, b, respectively. Median PFS was 39 months (95%CI: 21-not reached) for FCB and 28 months (95%CI:10-not reached) for Flu-Bu. Median OS was not reached for FCB and was 54 months (95%CI: 15-not reached) for Flu-Bu. Estimated 3-year PFS probabilities were 52% (95%CI:44–62%) for FCB and 48% (95%CI:41–58%) for Flu-Bu. Estimated 3-year OS probabilities were 57% (95%CI: 49–67%) for FCB and 53% (95%CI: 45–62%) for Flu-Bu. The median GVHD-free, relapse-free survival (GRFS) for FCB was 9.7 months (95%CI: 7.8–15.8), and for the Flu-Bu group, it was 9.1 months (95%CI: 6.9–11.1), *p* = 0.896.

### Relapse and non-relapse mortality (NRM)

Cumulative 100-day NRM estimates were 5.0% (95%CI: 2–10%) for FCB and 2.3% (95%CI: 0.6–6.1%) for Flu-Bu. The cumulative one- and 3-year NRM estimates were 16.7% (95%CI: 11–24%) and 22.6% (95%CI:16–30.2%) for FCB and 10.0% (95%CI:5.6–16%) and 12.3% (95%CI: 6.5–19%) for Flu-Bu.

The cumulative 1- and 3-year relapse incidences (RI) were 18% (95%CI: 12–26%), and 25% (95%CI: 18–33%), respectively, for those treated with FCB and 35% (95%CI: 26–43%) and 39% (95%CI: 31–48%), respectively, for Flu-Bu (*p* = 0.02). When RI was evaluated according to disease status at transplant, the benefits of FCB were larger for NCR patients, whose 1-year and 3-year RI for the FCB group ≤60 years were 22% (95%CI: 10–37%) and 34% (95%CI: 19–49%), respectively, compared with 50% (95%CI: 33–65%) and 56% (95%CI: 38–70%), respectively for the Flu-Bu group (*p* = 0.037). For NCR patients older than 60, the difference was even larger, the FCB group had 1- and 3-year RI of 5.0% (95%CI: 0.3–21%) and 10.0% (95%CI: 1.5–28%), respectively, versus 52% (95%CI: 31–70%) and 56% (95%CI: 34–73%), respectively, for the Flu-Bu group (*p* = 0.003).

**Fig. 2:**
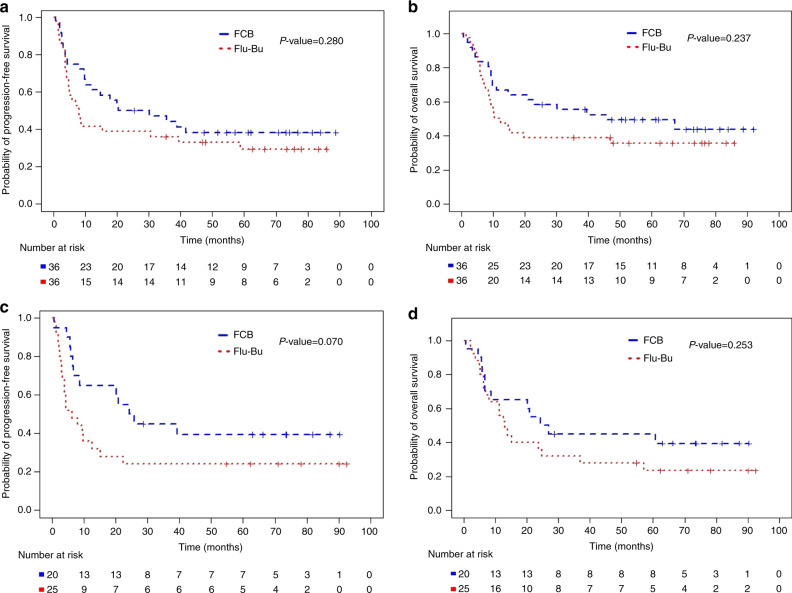
Progression-Free and Overall Survival of all Patients with active disease, NCR. **a** PFS for patients transplanted in NCR (Diagnosis = NCR, Age ≤ 60) Kaplan–Meier plots of PFS for patients treated with FCB (Number of patients = 36, number of PFS events =22, median PFS 25.3 months) and Flu-Bu (Number of patients = 36, number of PFS events =25, median PFS 8.1 months). The *P* value 0.280 is from the log-rank test. **b** Overall survival for the patients transplanted with active disease, NCR age ≤60 years. Kaplan–Meier plots of OS for patients treated with FCB (Number of patients = 36, number of deaths = 19, median OS 45.6 months) and Flu-Bu (Number of patients =36, number of deaths = 23, median OS 11.4 months). The *P* value 0.237 is from the log-rank test. **c** Subgroup (Diagnosis = NCR, Age > 60) Kaplan–Meier plots of PFS for patients treated with FCB (Number of patients = 20, number of PFS events = 12, median PFS 25.0 months) and Flu-Bu (Number of patients = 25, number of PFS events = 19, median PFS 6.4 months). The *P* value 0.070 is from the log-rank test. **d** Subgroup active disease, NCR, age >60 years. Kaplan–Meier plots of OS for patients treated with FCB (Number of patients = 20, number of deaths = 12, median OS 25.6 months) and Flu-Bu (Number of patients = 25, number of deaths = 19, median OS 13.0 months). The *P* value 0.253 is from the log-rank test.

**Fig. 3:**
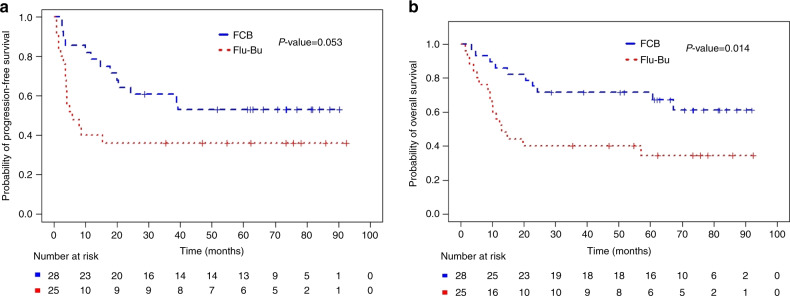
Progression-Free and Overall Survival of all Patients with active disease, NCR, and low HCT-CI (0-2). **a** Subgroup (HCT-CI 0-2, NCR) Kaplan–Meier plots of PFS for FCB (Number of patients = 28, number of PFS events = 13, median PFS not reached) and Flu-Bu (Number of patients = 25, number of PFS events = 16, median PFS 5.8 months). The *P* value 0.053 is from the log-rank test. **b** Subgroup (HCT-CI 0-2, NCR) Kaplan–Meier plots of OS for FCB (Number of patients = 28, number of deaths=10, median OS not reached) and Flu-Bu (Number of patients = 25, number of deaths = 16, median OS 13.0 months). The *P* value 0.014 is from the log-rank test.

The large observed relapse benefit for FCB was offset by higher NRM Fig. 2a, b, and only the NCR group above age 60 maintained its PFS and OS benefit (Fig. 2c, d). At the time of study design, it had not been conclusively shown whether HCT-CI predict post-transplant outcome if patients’ KPS and other eligibility criteria are acceptable, but today this is considered likely. We thus examined the NCR subgroups with HCT-CI (0–2) versus higher [3–10]. Patients with lower HCT-CI (0–2) had the highest benefit from FCB for both PFS and OS, illustrated in Fig. 3a, b, respectively.

### Covariate-adjusted analyses

Fitted Bayesian PE regression models for PFS are presented in Table 3 for the additive main effects model and Table S2 for the model including treatment-covariate interactions. While PBE = 0.67 for the FCB versus Flu-Bu effect in the main effects model (Table 3), the model accounting for treatment-covariate interactions had PBE = 0.94 for FCB (Table S2). Similarly structured regression models for OS, presented in Tables S3, S4, are consistent with those seen for PFS. The disagreement between the additive model and interaction model estimates of the FCB versus Flu-Bu effects is dueto the fact that in the additive model estimated effects are averages over subgroups within which the FCB-vs- Flu-Bu effects go in opposite directions [29]. For example, regardless of age, the subgroups with [HCT-CI = 0–2, NCR] have PBE = 0.92–0.98 for FCB vs Flu-Bu, while the subgroups with [HCT-CI ≥ 3, CR] have PBE = 0.07 to 0.13 for FCB vs Flu-Bu (Table S5). This implies FCB is more desirable than Flu-Bu for patients with [HCT-CI = 0–2, NCR] and less desirable for patients with [HCT-CI ≥ 3, CR]. These results suggest that attention should be paid to individual patient disease status and comorbidities when choosing treatment [16].

**Table 3:** Fitted Bayesian additive piecewise exponential model for PFS time (Number of patients = 249, number of PFS events = 130).

Variable	Reference	Posterior quantities			
		Mean effect	Standard deviation	95% credible intervals	Probability of a beneficial effect = Pr(*β* < 0|Data)
Treatment FCB	Flu-Bu	−0.08	0.18	−0.44, 0.26	0.67
Age (≤60)	>60	−0.38	0.19	−0.75, 0.00	**0.97**
Donor Relation (Unrelated)	Sibling	0.15	0.22	−0.28, 0.58	0.24
Diagnosis (AML)	MDS	0.32	0.22	−0.11, 0.77	**0.07**
Cytogenetic risk group (Intermediate/Good)	Poor	−0.42	0.18	−0.77, −0.07	**0.99**
CR	NCR	−0.87	0.20	−1.26, −0.47	**1.00**
Cell Type (HPC_A)	HPC-M	−0.18	0.22	−0.61, 0.24	0.80
HCT-CI score (≥3)	0–2	0.60	0.20	0.23, 0.98	**0.00**

**Fig. 4:**
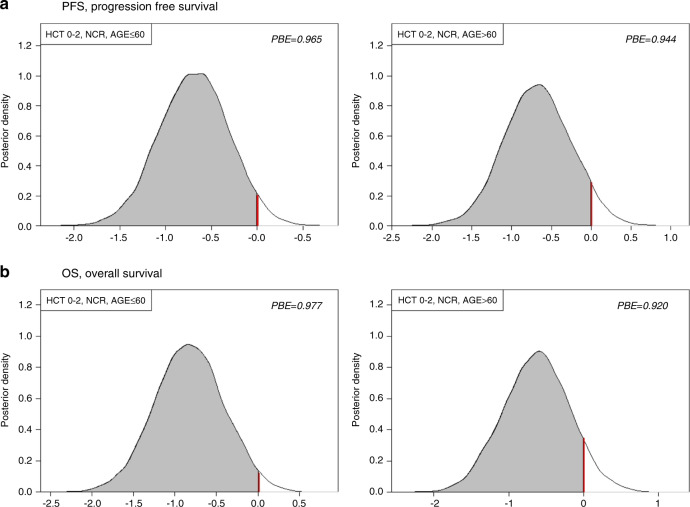
Bayesian density plots describing the probability for a beneficial outcome for the NCR subgroups <60 and >60 years and with a HCT-CI 0–2. **a** Progression-free survival (PFS), and **b** Overall survival (OS).

Kaplan–Meier plots for the subgroup of patients with HCT-CI (0–2) and NCR are presented in Fig. 3a, b. Corresponding Bayesian posterior plots for PFS and OS are presented in Fig. 4. Additional subgroup-specific comparisons showed no meaningful between-treatment effect on PFS in the [AML, CR] subgroup (*p* = 0.74, log-rank test), the [AML, NCR] subgroup (*p* = 0.57, log-rank test), or in MDS patients (*p* = 0.23, log-rank test), with similar non-significant differences for OS. While FCB patients were at higher risk for developing grades II-IV aGVHD and cGVHD (HR = 1.46, 95%CI 0.98–2.19) their risk for leukemic progression (HR = 0.91, 95%CI 0.61–1.36) was lower, Table S6.

## Discussion

This randomized phase III trial showed an acceptable toxicity profile and better disease control with FCB in patients who had active disease at start of pretransplant conditioning, but no overall difference in either PFS or OS between the two treatment groups. Patients with HCT-CI of ≥3 were at increased risk of treatment-related serious adverse events, and associated mortality, with the FCB regimen.

Improved antileukemic efficacy was seen when Flu was partly substituted with Clo in combination with Bu as conditioning for AML and MDS patients, in keeping with reported cell line data [9, 10]. The immunosuppressive effects of FCB allowed consistent engraftment, there were no graft failures in the 118 evaluable FCB patients, as seen previously [11]. The 100-day 5% NRM in patients with 0–10 comorbid conditions after FCB, compared with a 2.3% NRM after Flu-Bu, was similar to that previously reported [3, 4, 8]. Toxicity and adverse event profiles also were also comparable for the two regimens (Table 2). While both Bu and Clo are hepatotoxic, there was an analogous incidence of regimen-related bilirubin elevation in the two treatment arms (Table 2). For VOD/SOS, there was no case after Flu-Bu, while three of 120 patients with high HCT-CI in the FCB arm developed this complication, one of whom died. While more serious infections, grades 3–5, were observed after FCB (Table 2), there was no difference in the incidence of lethal infections between the two age groups, and all patients who died had high HCT-CI scores (median 5, range 3–10).

Clofarabine’s estimated average β-T_½_ is 6–7 h(range, 3.5–18.7 h), [30], reminiscent of the terminal half-life of about 8.5 reported for fludarabine (range, 3.7–22 h) [31]. However, Clo is nephrotoxic [32], which may prolong clearance of both Clo and Flu in patients with borderline low creatinine clearance. Thus, the higher incidence of infections after FCB may be due to decreased renal NA clearance in some patients when two NAs are utilized together, causing cytotoxic stress on the incoming graft with delayed post-transplant immune reconstitution. This may be manifested as an increased risk for serious post-transplant infections, analogous to that reported by Long-Boyle [31]. Going forward, we will implement three rather than two rest days between Flu/Clo and graft infusion, and assay Flu and Clo in plasma on days −1 and 0 to discern whether some patients indeed have altered clearance of the respective NAs [33].

It may be argued that the apparent lack of improvement in PFS/OS with FCB compared with Flu-Bu for the entire population (Fig. 1a, b) demonstrated a lack of benefit of Clo, and therefore Flu-Bu should remain standard of care [34]. While this may be true for patients transplanted in MRD-negative CR, different AML subpopulations may have varying sensitivities to the cytotoxic agents, exemplified by NCR patients, who also have a larger disease bulk. This may explain the 1- and 3-year RIs of 18% and 25% after FCB vs. 35% and 39% after Flu-Bu. Since the entire sample is a mixture of CR and NCR patients, overall FCB-vs- Flu-Bu effect estimates will obscure benefits obtained with FCB in NCR subgroups (Figs. 1a, b, 2a–d). The increased synergy between two NAs and Bu compared with one NA and Bu likely will extract a clinical toll in the form of normal organ stress (adverse events), which was more pronounced with FCB than with Flu-Bu, especially in patients with high HCT-CI [16]. After stratifying for CR/NCR, there was little benefit for PFS or OS in CR patients conditioned with FCB (Fig. 1a, b), but a substantive advantage for FCB in the NCR group (Fig. 2a–d). In the overall population, this was seen as a longer time from transplant to relapse or death after FCB. It could also be argued that the 3-year RI of ~40% in the NCR group after Flu-Bu is high compared with recent literature data [35]. However, at MD Anderson non-transplant therapies traditionally have been favored, which produces a patient pool with high-risk features, commonly including relapsed/refractory disease. The NCR group included patients with >5% blasts and/or extramedullary disease, with no exclusion for a high marrow and/or peripheral blood blast count. A large fraction of NCR patients had bulky disease with both marrow involvement and high circulating blasts. These factors may help to explain the seemingly high relapse rate, which for the Flu-Bu group was similar to that reported by de Lima [4]. This scenario was complicated by allowing patients with high HCT-CI [16], independent of end-organ function or KPS. While subgroup-specific inferences may be considered post hoc, the trial protocol specified that treatment subgroup effects should be investigated if warranted by goodness-of-fit analyses. Comorbidity score, disease status, and age are today well-established prognostic covariates that, due to the nature of the FCB and Flu-Bu regimens, may be considered a priori likely to interact with these treatments. When examining effects on patients with active AML and low HCT-CI (0–2), FCB confers a major advantage over Flu-Bu in this [NCR, HCT-CI = 0–2] subgroup; while median PFS and OS times after FCB had not been reached at 5 years post-transplantation, the corresponding medians after Flu-Bu were 5.8 and 13.0 months, respectively (Fig. 3a, b). These findings suggest that confirmatory trials of FCB in patients with low HCT-CI scores and detectable disease at the start of pretransplant conditioning should be conducted.

The Bayesian analyses identified the contribution of higher HCT-CI to the observed differences (Table 3, Tables S2–S5, Fig. 4). The slightly higher intensity of FCB led to higher NRM in patients with HCT-CI ≥ 3 in the FCB group compared with the Flu-Bu cohort, partly obscuring the antileukemic benefit of FCB. While FCB may exert a profoundly better antileukemic effect than Flu-Bu in some patients, it should be incorporated into a carefully personalized treatment program and used with caution in patients with higher HCT-CI. In patients who are generally fit, incorporating the need for HCT-CI 0–2, FCB was well tolerated up to age 70 without apparent need to adjust the doses of Flu or Clo, with the caveat of considering reducing Bu to an average daily Bu-SE of 4000 µMol-min for patients above age 60, as was done in this study.

## Conclusions

There was no meaningful difference between FCB and Flu-Bu in either PFS or OS for the entire population, but a substantive superiority of FCB in NCR patients or patients with age ≤60. However, while remission status and HCT-CI today are well-established prognostic variables, HCT-CI was not prospectively validated at the time of this study’s design. Inferences regarding predictive effects of FCB vs. Flu-Bu in subgroups defined by these covariates should be considered non-confirmatory due to the possibility of bias due to post-hoc subgroup selection. This may be investigated in a future study to obtain confirmatory results, and application of FCB should be personalized to account for whether the patient is in CR/NCR, and by including assessment of the risk for treatment-related complications based on the patient’s HCT-CI and PS. Finally, FCB can be utilized in appropriate patients up to 70 years of age, and may yield better disease control than Flu-Bu over time.

## Supplementary information

Supplemental Files

## Data Availability

The datasets generated during and/or analyzed during the current study are available from the corresponding author on reasonable request.
