# Supplementary material for: A randomized phase III study of pretransplant conditioning for AML/MDS with fludarabine and once daily IV busulfan ± clofarabine in allogeneic stem cell transplantation
Source: Bone Marrow Transplant. 2022 May 24;57(8):1295–303. doi: 10.1038/s41409-022-01705-7 (PMC9352570; doi:10.1038/s41409-022-01705-7)
Supplement: Supplementary file 1 — Supplemental Files [file 41409_2022_1705_MOESM1_ESM.docx]

**Supplemental Materials**

1. **TABLES**

**Table S1:** Summary of engraftment, aGVHD and cGVHD, overall and by treatment group.

| Variable | N (evaluable) | Overall | Flu-Bu | FCB |
| --- | --- | --- | --- | --- |
| ***Chimerism, n (%)** | 248 |  |  |  |
| Full Donor |  | 134 (54.0) | 63 (48.5) | 71 (60.2) |
| Mixed chimerism |  | 114 (46.0) | 67 (51.5) | 47 (39.8) |
| ****Best Chimerism, n (%)** | 248 |  |  |  |
| Full Donor |  | 221 (89.1) | 112 (86.2) | 109 (92.4) |
| Mixed chimerism |  | 27 (10.9) | 18 (13.8) | 9 (7.6) |
| **Time to engraftment, Days, median (SD)**  **min-max** | 248 | 12.5 (2.7)    10-25 | 12.5 (2.4)  10-23 | 12.6 (3.1)  10-25 |
| **Time to PLT20, Days,**  **Median (SD)**  **min-max** | 239 | 15.6 (8.2)  0-67 | 15.2 (6.9)  0-46 | 16.0 (9.5)  1-67 |
| **Time to PLT50, Days, Median (SD)**  **min-max** | 228 | 24.5 (28.8)  8-342 | 22.0 (14.3)  9-98 | 27.3 (39.1)  8-342 |
| **^#^Disease Status, n (%)** | 246 |  |  |  |
| CR |  | 240 (97.6) | 124 (96.1) | 116 (99.1) |
| NR = No Response/ Progr. Dx |  | 6 (2.4) | 5 (3.9) | 0 (0.0) 1 (0.9) |
|  |  |  |  |  |
| **Acute GVHD, n (%)** | 248 |  |  |  |
| No |  | 78 (31.5) | 50 (38.5) | 28 (23.7) |
| Yes |  | 170 (68.5) | 80 (61.5) | 90 (76.3) |
| **Acute GVHD Max, n (%)** | 248 |  |  |  |
| 0-1 |  | 137 (55.2) | 79 (60.8) | 58 (49.2) |
| 2 |  | 95 (38.3) | 43 (33.1) | 52 (44.1) |
| 3 |  | 11 (4.4) | 6 (4.6) | 5 (4.2) |
| 4 |  | 5 (2.0) | 2 (1.5) | 3 (2.5) |
| **Chronic GvHD, n (%)** | 236 |  |  |  |
| No |  | 143 (60.6) | 78 (62.4) | 65 (58.6) |
| Yes |  | 93 (39.4) | 47 (37.6) | 46 (41.4) |

* Evaluated at one month post-transplant by fractionated T-cell chimerism.

**Evaluated at any time post-transplant.

^#^ Evaluated at one month post-transplant

**Table S2.** Fitted Bayesian piecewise exponential model for PFS including interaction terms FCB x Age60, FCB x CR, and FCB x [HCT-CI score ≥3] (Number of patients=249, number of deaths=130).

| **Variable** | **Reference** | **Posterior quantities** | | | |
| --- | --- | --- | --- | --- | --- |
|  |  | **Mean Effect** | **Standard deviation** | **95%**  **credible**  **intervals** | **Probability**  **of a beneficial effect =**  **Pr(β < 0 \| Data)** |
| **Treatment**  (FCB) | Flu-Bu | -0.673 | 0.424 | -1.498, 0.156 | **0.944** |
| **Younger Age**  (≤60) | >60 | -0.384 | 0.268 | -0.904, 0.145 | **0.923** |
| **Donor Relation**  (Unrelated) | Sibling | 0.094 | 0.220 | -0.345, 0.523 | 0.332 |
| **Diagnosis**  (AML) | MDS | 0.303 | 0.223 | -0.131, 0.744 | **0.086** |
| **Cytogenetic risk group**  (Intermediate/Good ) | Poor | -0.478 | 0.181 | -0.828, -0.121 | **0.996** |
| **CR**  (CR) | NCR | -1.123 | 0.278 | -1.676, -0.583 | **1.000** |
| **Cell Type**  (HPC_A) | HPC-M | -0.207 | 0.217 | -0.634, 0.215 | 0.830 |
| **HCT-CI score**  (≥3) | 0-2 | 0.289 | 0.265 | -0.213,0.820 | 0.138 |
| **Treatment x Age**  (FCB × Age60) | >60 | -0.015 | 0.389 | -0.774, 0.761 | 0.519 |
| **Treatment x CR**  (FCB × CR) | NCR | 0.480 | 0.388 | -0.280, 1.255 | 0.108 |
| **Treatment x HCT-CI score**  (FCB × ≥3) | 0-2 | 0.608 | 0.391 | -0.146,1.385 | **0.058** |

**Table S3.** Fitted Bayesian additive piecewise exponential model for OS time (number of patients=249, number of deaths=123).

| **Variable** | **Reference** | **Posterior quantities** | | | |
| --- | --- | --- | --- | --- | --- |
|  |  | **Mean effect** | **Standard**  **deviation** | **95%**  **credible**  **intervals** | **Probability**  **of a beneficial effect**  **Pr(β < 0 \| Data)** |
| **Treatment**  (FCB) | Flu-Bu | 0.026 | 0.185 | -0.335, 0.388 | 0.444 |
| **Age**  (≤60) | >60 | -0.483 | 0.196 | -0.861, -0.096 | **0.992** |
| **Donor Relation**  (Unrelated) | Sibling | 0.223 | 0.222 | -0.205, 0.662 | 0.155 |
| **Diagnosis**  (AML) | MDS | 0.331 | 0.227 | -0.106, 0.778 | 0.073 |
| **Cytogenetic risk group**  (Intermediate/Good) | Poor | -0.520 | 0.182 | -0.876, -0.166 | **0.998** |
| **CR**  (CR) | NCR | -0.772 | 0.205 | -1.170, -0.369 | **1.000** |
| **Cell Type**  ^#^(HPC-A) | *HPC-M | -0.130 | 0.218 | -0.548, 0.303 | 0.726 |
| **HCT-CI score**  (≥3) | 0-2 | 0.684 | 0.201 | 0.297, 1.080 | **0.000** |

^#^(HPC-A); Progenitor cells obtained through an apheresis procedure of peripheral blood.

*HPC-M; Bone Marrow progenitor cells.

**Table S4.** Fitted Bayesian piecewise exponential model for OS including the FCB x Age60, FCB x CR, and FCB x [HCT-CI score ≥3], (Number of patients=249, number of deaths=123).

| **Variable** | **Reference** | **Posterior quantities** | | | |
| --- | --- | --- | --- | --- | --- |
|  |  | **Mean Effect** | **Standard deviation** | **95%**  **credible**  **intervals** | **Probability**  **of a beneficial effect**  **Pr(β < 0 \| Data)** |
| **Treatment**  (FCB) | Flu-Bu | -0.628 | 0.449 | -1.506, 0.253 | **0.920** |
| **Younger Age**  (≤60) | >60 | -0.404 | 0.282 | -0.947, 0.163 | **0.924** |
| **Donor Relation**  (Unrelated) | Sibling | 0.165 | 0.232 | -0.287, 0.626 | 0.239 |
| **Diagnosis**  (AML) | MDS | 0.280 | 0.235 | -0.172, 0.748 | 0.115 |
| **Cytogenetic risk group**  (Intermediate/Good) | Poor | -0.600 | 0.189 | -0.970, -0.228 | **0.999** |
| **Diagnosis**  (CR) | NCR | -0.998 | 0.285 | -1.554, -0.435 | **1.000** |
| **Cell Type**  (HPC_A) | HPC-M | -0.162 | 0.224 | -0.595, 0.284 | 0.769 |
| **HCT-CI score**  (≥3) | 0-2 | 0.256 | 0.268 | -0.258, 787 | 0.171 |
| **Treatment x Age**  (FCB × Age60) | >60 | -0.197 | 0.407 | -1.000, 0.598 | 0.689 |
| **Treatment x CR**  (FCB × CR) | NCR | 0.358 | 0.400 | -0.430, 1.143 | 0.182 |
| **Treatment x HCT-CI score**  (FCB × ≥3) | 0-2 | 0.929 | 0.424 | 0.094, 1.768 | **0.014** |

**Table S5.** FCB versus Flu-Bu comparisons in subgroups defined by HCT, Age60, and disease status (CR vs NCR). Estimated FCB effects are based on the fitted Bayesian piecewise exponential model for OS (Table 6) with interaction terms FCB x Age60, FCB x CR, FCB x [HCT-CI score > 3], (number of patients=249, number of deaths=123).

| **Subgroup** | **Posterior quantities** | | | |
| --- | --- | --- | --- | --- |
|  | **Mean effect** | **Standard**  **deviation** | **95%**  **credible**  **interval** | **Probability**  **of a beneficial effect**  **Pr(β < 0 \| Data)** |
| HCT-CI 0-2; CR, AGE≤60 | -0.345 | 0.572 | -1.468, 0.769 | 0.727 |
| HCT-CI 0-2; CR, AGE>60 | -0.270 | 0.527 | -1.323, 0.751 | 0.692 |
| HCT-CI 0-2; NCR, AGE≤60 | -0.825 | 0.416 | -1.649, -0.014 | **0.977** |
| HCT-CI 0-2; NCR, AGE>60 | -0.628 | 0.449 | -1.506, 0.253 | **0.920** |
| HCT-CI ≥3; CR, AGE≤60 | 0.584 | 0.519 | -0.413, 1.608 | **0.130** |
| HCT-CI ≥3; CR, AGE>60 | 0.659 | 0.436 | -0.197, 1.519 | **0.066** |
| HCT-CI ≥3; NCR, AGE≤60 | 0.104 | 0.341 | -0.560, 0.773 | 0.377 |
| HCT-CI ≥3; NCR, AGE>60 | 0.301 | 0.357 | -0.401, 0.994 | 0.199 |

**Table S6.** Associations between grade II acute GVHD and PFS from landmark Cox models starting at day 100, adjusting treatment of FCB versus Flu-Bu (n=216). Patients who died or progressed within 100 days were excluded from this analysis, and those who experienced grade II acute GVHD >100 days were treated as non-acute GVHD patients.

| **Variable** | **Hazard ratio (HR)** | **95% Confidence**  **Interval for HR** | **P-value** |
| --- | --- | --- | --- |
| **Acute GVHD,**  Grade 2 | 1.46 | (0.98, 2.19) | 0.065 |
| **Treatment,**  FCB | 0.91 | (0.61, 1.36) | 0.641 |
